# Supplementary material for: Ferroelectric solitons crafted in epitaxial bismuth ferrite superlattices
Source: Nat Commun. 2023 Jul 13;14:4178. doi: 10.1038/s41467-023-39841-3 (PMC10345089; doi:10.1038/s41467-023-39841-3)
Supplement: Supplementary file 1 — Supplementary Information [file 41467_2023_39841_MOESM1_ESM.pdf]

Supplementary Information:

Ferroelectric Solitons Crafted in Epitaxial Bismuth Ferrite Superlattices

Vivasha Govinden[1,11], Peiran Tong[2,11], Xiangwei Guo[3,4,5,11], Qi Zhang[1], Sukriti Mantri[6], Mohammad Moein Seyfouri[1,7], Sergei Prokhorenko[6], Yousra Nahas [6], Yongjun Wu[3,5], Laurent Bellaiche[6], Tulai Sun[2,8], He Tian[2,9,*], Zijian Hong[3,5,*], Nagarajan Valanoor[1,*], and Daniel Sando[1,10,*]

[1]*School of Materials Science and Engineering, University of New South Wales Sydney, Kensington, New South Wales, Australia*

[2]*Center of Electron Microscopy, School of Materials Science and Engineering, State Key Laboratory of Silicon Materials, Zhejiang University, Hangzhou, Zhejiang, China*

[3]*School of Materials Science and Engineering, Zhejiang University, Hangzhou, Zhejiang, China*

[4]*Institute of Advanced Semiconductors & Zhejiang Provincial Key Laboratory of Power Semiconductor Materials and Devices, Hangzhou Innovation Center, Zhejiang University, Hangzhou, Zhejiang, China*

[5]*Cyrus Tang Center for Sensor Materials and Applications, State Key Laboratory of Silicon Materials, Zhejiang University, Hangzhou, Zhejiang, China*

[6]*Physics Department and Institute for Nanoscience and Engineering, University of Arkansas, Fayetteville, AR, USA*

[7]*Solid State and Elemental Analysis Unit, Mark Wainwright Analytical Centre, University of New South Wales, Sydney, Australia*

[8]*Center for Electron Microscopy, State Key Laboratory Breeding Base of Green Chemistry Synthesis Technology and College of Chemical Engineering, Zhejiang University of Technology, Hangzhou, Zhejiang, China*

[9]*School of Physics and Microelectronics, Zhengzhou University, Zhengzhou, Henan, China*

[10]*School of Physical and Chemical Sciences, University of Canterbury, Christchurch, New Zealand*

[11]*These authors contributed equally.*

*Correspondence to: hetian@zju.edu.cn, hongzijian100@zju.edu.cn, nagarajan@unsw.edu.au, daniel.sando@canterbury.ac.nz

Supplementary Note 1. Layer-by-layer growth of $BiFeO_3$ (BFO) on $LaAlO_3$ (LAO)

Here, we show evidence for high-quality layer-by-layer growth of T-like BFO on LAO substrates, using our unique growth chamber. The large target-substrate distance of ~10 cm results in low growth rate and a slightly modified stoichiometry, where Bi_2O_3 nano-pockets form randomly throughout the film and maintain strain coherence. This allows us to fabricate T-like BFO with no mixed phase to thicknesses up to and beyond 60 nm[1]. Figure S1a shows *in-situ* reflection high-energy electron diffraction (RHEED) oscillations for BFO//LAO (001), which persist for several 10s of unit cells. The Laue oscillations around the 001 BFO peak in X-ray diffraction scans (Fig. S1b) attest to a highly coherent film with smooth interfaces, and the *ex-situ* AFM images (Fig. S1c,d) show an extremely smooth surface with atomic steps, consistent with the 2D RHEED pattern observed after growth (bottom right inset of Fig. S1a).

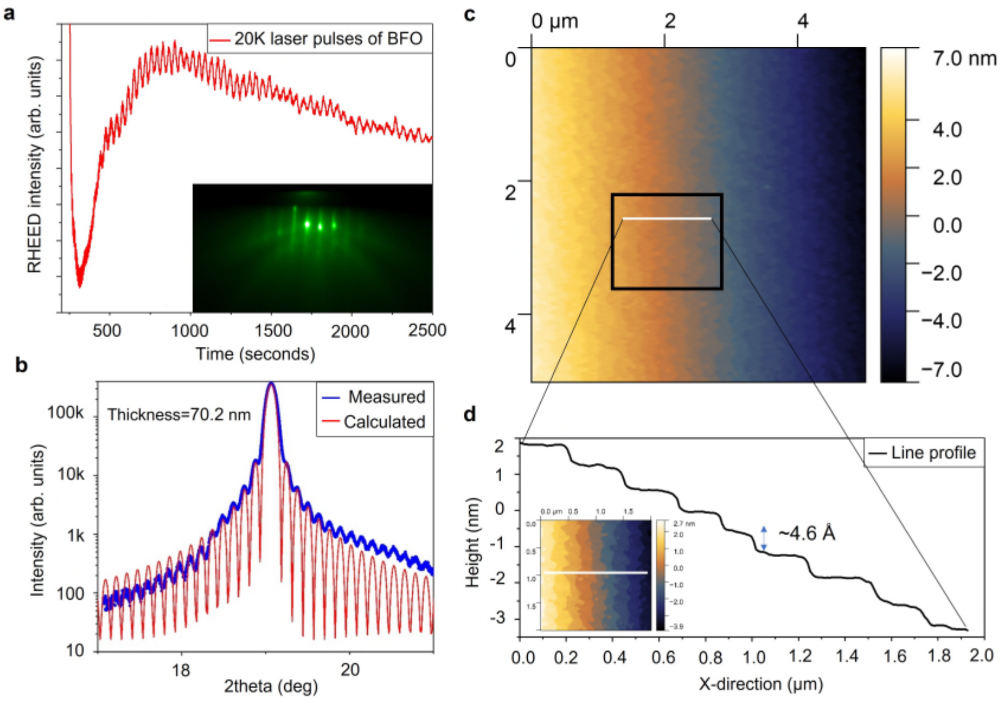

Fig. S1 | Layer-by-layer growth of $BiFeO_3$ (BFO). (**a**) Reflection high-energy electron diffraction (RHEED) specular spot intensity during BFO deposition on (001) LAO substrate. The bottom right inset shows the RHEED diffraction pattern after deposition. (**b**) X-ray diffraction 2θ-ω scan near the 001 BFO reflection showing clear Laue oscillations. (**c**) Topography of single-layered BFO grown on LAO (001) acquired by atomic force microscopy, and (**d**) the scan at higher magnification allows to determine step height of ~4.6 Å. Note that this dataset does not represent the samples discussed in the main paper, it serves only to prove our capacity to produce high quality layer-by-layer BFO on LAO.

Supplementary Note 2. PFM characterization of various superlattice combinations

In this supplementary note, we present piezoresponse force microscopy (PFM) images of $(BFO_m/STO_n)_{10}$ (where STO stands for $SrTiO_3$) superlattices with different thicknesses *m* and *n*. Figure S2 presents the domain configurations in various BFO/STO superlattices. Bubble-shaped domains were observed for $(BFO_8/STO_4)_{10}$ and $(BFO_7/STO_4)_{10}$ samples, with domain sizes ranging from approximately 10 nm to 20 nm (Fig. S2a, b, e and f). Smaller domains were observed for the $(BFO_7/STO_4)_{10}$ superlattice. Upon decreasing the BFO and STO layer thicknesses to $m = 5$ and $n = 2$ u.c., respectively, a coexistence of labyrinthine and bubble-shaped domains was observed (Fig. S2c and e). Finally, when the STO dielectric layer is thicker than the BFO ferroelectric layer (i.e., $m = 2$, $n = 4$), a monodomain state with no topological solitons was observed (Fig. S2d and h).

We thus found using this optimization process that the ideal configuration is $m = 7$, $n = 4$, which provides the perfect strain and electrical boundary conditions to produce polar solitons. This superlattice structure is the one described extensively in the main manuscript.

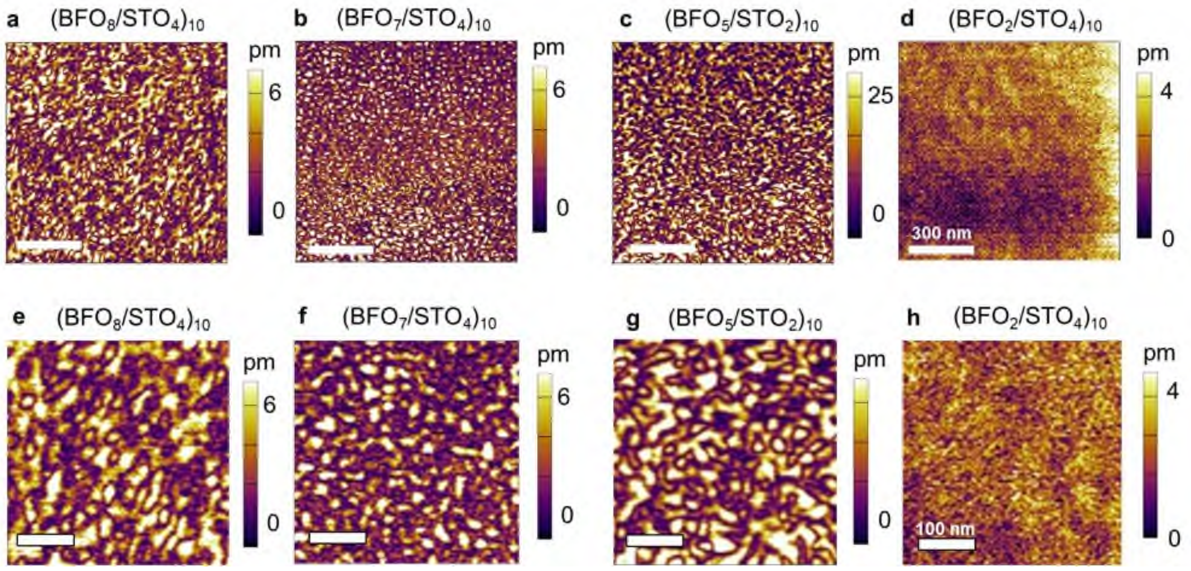

Fig. S2 | Domain configurations in BFO/STO superlattice heterostructures with varying layer thicknesses. Piezoresponse force microscopy amplitude images for (**a**) and (**e**) $(BFO_8/STO_4)_{10}$, (**b**) and (**f**) $(BFO_7/STO_4)_{10}$, (**c**) and (**g**) $(BFO_5/STO_2)_{10}$, (**d**) and (**h**) $(BFO_2/STO_4)_{10}$. Scale bars in (**a-d**) and (**e-h**) are 300 nm and 100 nm, respectively.

Supplementary Note 3. Atomic resolution energy dispersive x-ray spectroscopy analysis

Here, we provide evidence for high quality superlattice structures with minimal chemical intermixing or diffusion. We prepared a cross-sectional sample of a $(BFO_7/STO_4)_{10}$ superlattice and performed energy-dispersive x-ray spectroscopy (EDS) analysis. The atomic resolution EDS mapping (Fig. S3) shows atomically and chemically sharp heterointerfaces with no interdiffusion. Further high-resolution STEM images are presented in Fig. S3e – across a large region of the sample, the interfaces are sharp and show now evidence of diffusion. The ability to fabricate high-quality superlattices wherein the long-range in-plane compressive strain can be sustained along the [001] direction, is a key step for realizing polar topologies.

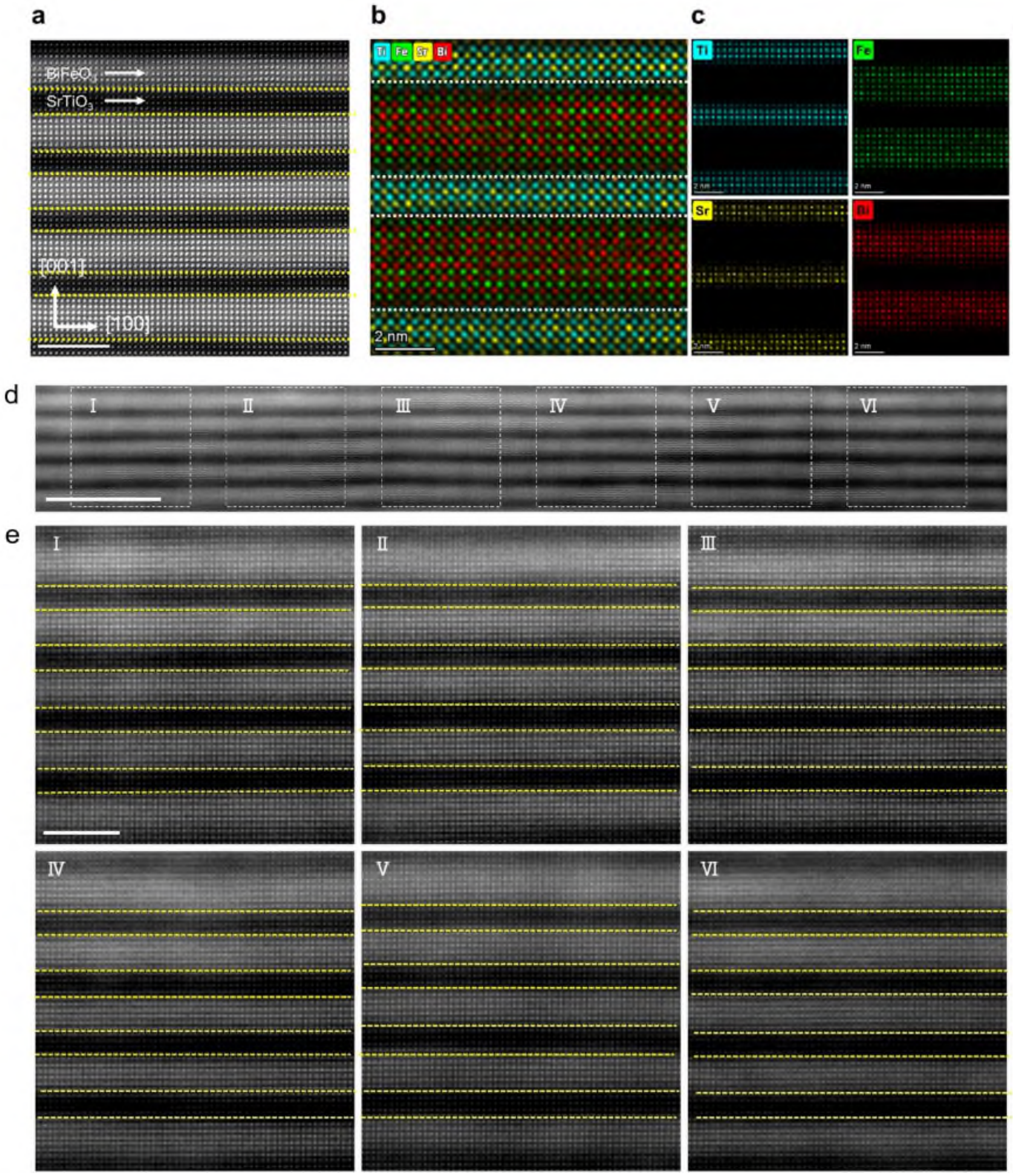

Fig. S3 | Structural characterization of a $(BFO_7/STO_4)_{10}$ superlattice. (**a**) Cross-sectional high-angle annular dark-field (HAADF)-STEM image. Dashed lines denote the interfaces between BFO and STO. Scale bar: 10 nm. (**b-c**), EDS-mapping of BFO/STO superlattice. Scale bar: 2 nm. (b) Overlapped EDS mapping of Ti, Fe, Sr, and Bi elements. (**c**) Individual Ti, Fe, Sr, and Bi element maps in the same location as shown in (b). (**d**) Low-magnification cross-section view of HAADF-STEM image of the BFO/STO superlattice. Scale bar, 20 nm. (**e**) Magnifications of areas labelled **I** through **VI** in panel (**d)**, showing sharp interfaces between BFO and STO. Scale bar, 5 nm.

Supplementary Note 4. X-ray diffraction (XRD) analysis

Here, we summarize the results of detailed x-ray studies on a $(BFO_7/STO_4)_{10}$ superlattice. Figure S4a shows that the experimental θ-2θ scan for the sample matches with a simulated XRD pattern obtained using a MATLAB program[2]. This custom-made MATLAB program uses layer thicknesses and out-of-plane lattice parameters as input variables. Fitting the experimental data to these simulated patterns allows the accurate measurement of the thickness and *c* lattice parameter of the individual layers depicted in the table in Fig. S4b. It confirms that the heterostructure consists of an 11 unit cell-thick bottom electrode of $La_{0.67}Sr_{0.33}MnO_3$ (LSMO), followed by 10 repeating units of 7 u.c. of BFO followed by 4 u.c. of STO, topped with a 7 u.c.-thick BFO capping layer. Note that the Laue oscillations shown in the simulated pattern are not observed in the experimental data likely due to the increased mosaicity and "disorder" induced by the topological soliton structures. These layer thicknesses are consistent with the results obtained from STEM images.

Figure S4c, d and e are asymmetrical reciprocal space maps about the (013), (103) and (113) reflections, respectively. The Q_x value for the film is smaller than that of the substrate, implying partial relaxation in the films. This fact is further confirmed by the in-plane lattice parameter of the film which is found to be 3.90 ± 0.01 Å, larger than the lattice parameter of the LAO substrate (3.79 Å). The out-of-plane lattice parameter of the film is found to be consistent for all reflections at 4.01 ± 0.01 Å. The lattice parameters derived from the reciprocal space maps are tabulated in Fig. S4f.

a

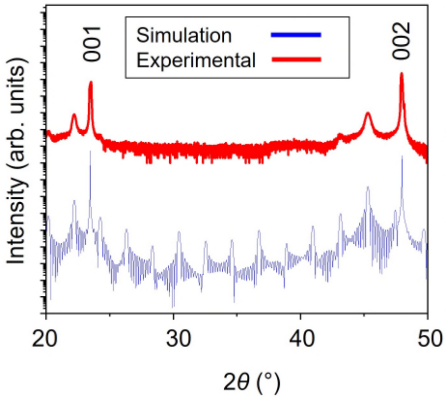

b

	# of unit cells (fitted)	*c* lattice parameter (fitted) (Å)	# laser pulses
LSMO	11	4.0	2500
BFO	7	4.0	1200
STO	4	4.0	500
BFO capping layer	7	4.0	1200

c **d**

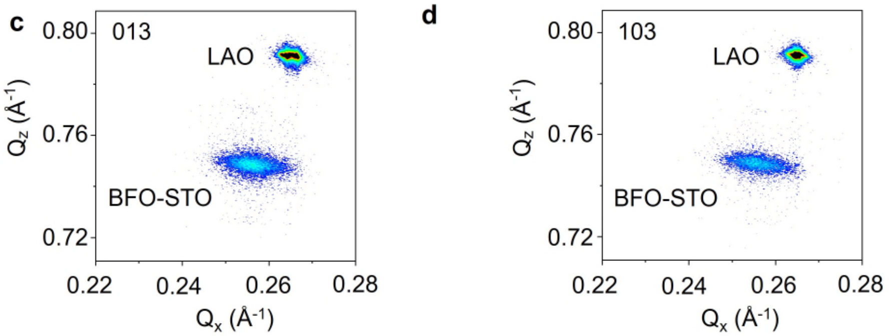

e

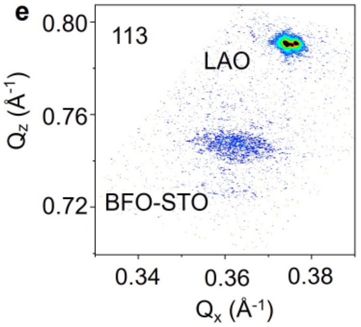

f

Reflection	Out-of-plane lattice parameter (Å)	Inplane lattice parameter (Å)
013	4.01	3.90
103	4.01	3.91
113	4.01	3.91

Fig. S4 | X-ray diffraction characterization of a ($BFO_7/STO_4)_{10}$ superlattice. (**a**) Experimental (red) and simulated (blue) θ-2θ scans depicting the 001 and 002 reflections. (**b**) Table with list of fitting parameters obtained from the MATLAB program. (**c-e**) Asymmetric reciprocal space maps near the (013), (103) and (113) reflections, respectively. (**f**) Tabulated lattice parameters calculated from the reciprocal space maps in (**c-e**).

Supplementary Note 5. Vector PFM analysis of solitons

Figure S5a-e shows the vertical PFM (V-PFM) and lateral PFM (L-PFM) of a centre divergence soliton structure in the BFO SL. The model (in Fig. S5a) depicts the topological defect polarization texture. At the centre and edge of the skyrmion, the dipoles have a strong out-of-plane component, separated by Néel-like walls. This Néel-like domain wall shows a strong in-plane component, pointing outwards from the core. Thus a strong in-plane amplitude is observed at the wall, with opposing phase contrast at the top and bottom hemispheres as shown by their respective arrows. Comparable results were obtained for the centre convergence soliton (Fig. S5f-j), with a convergent in-plane component for the Néel wall.

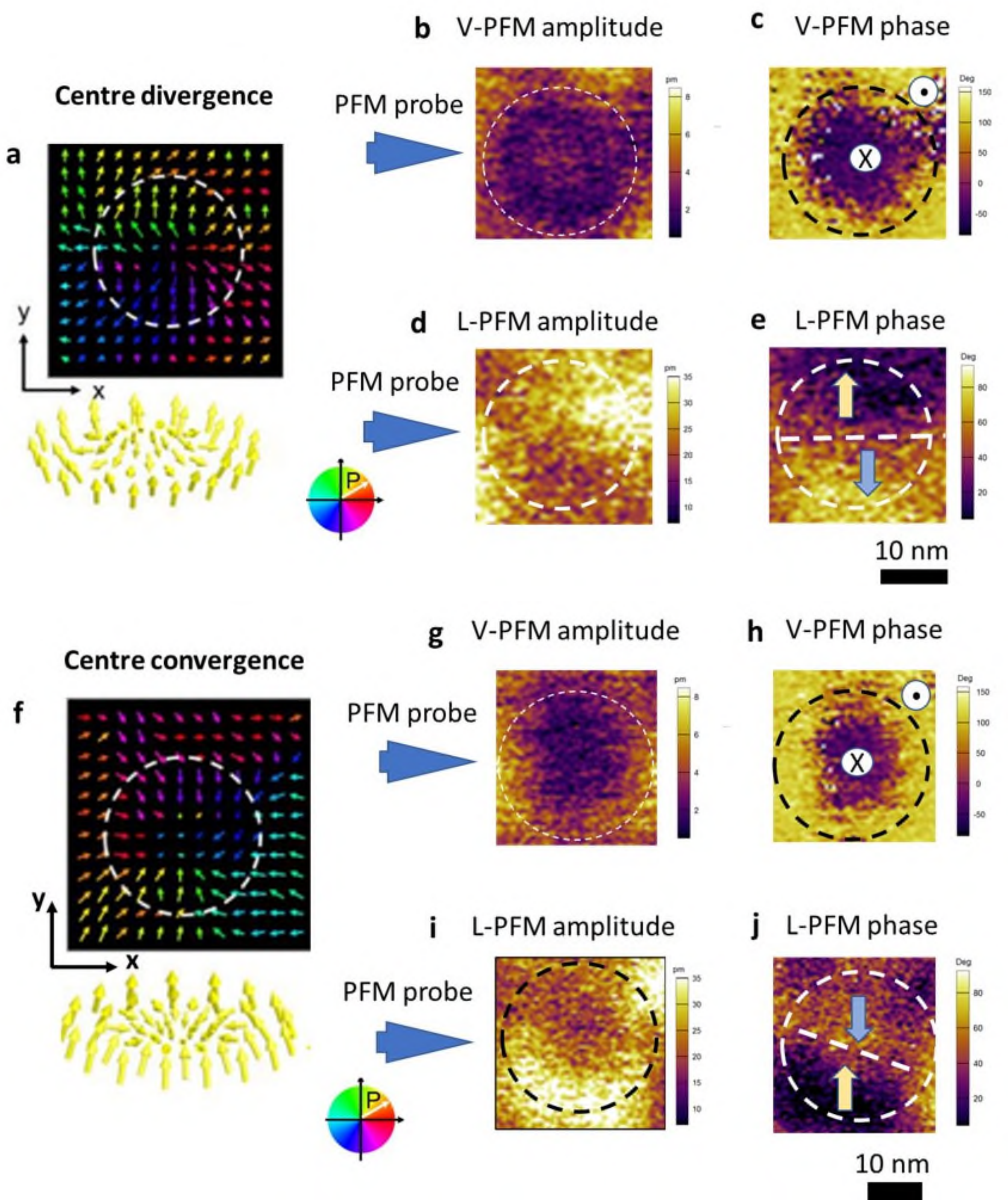

Fig. S5 | Vector PFM showing centre divergence and centre convergence configurations of solitons. (**a**) Planar TEM and 3-D model of centre divergence domain. Vertical (**b-c**) and lateral (**d-e**) PFM amplitude and phase of a centre divergence domain. (**f**) Planar TEM and 3-D model of centre convergence domain. Vertical (**g,h**) and lateral (**i,j**) PFM amplitude and phase of a centre convergence domain.

Supplementary Note 6. Details of polar displacement mapping, and evidence for widespread soliton existence

Polar displacement vector mapping analysis of HAADF-STEM images can image nanoscale polar structures. The planar and cross-sectional view HAADF-STEM images and corresponding polar maps (Fig. S6) provided detailed information on what the structures we observed in PFM are and indicated a 3D visualization of a variety of polar topologies in our superlattices.

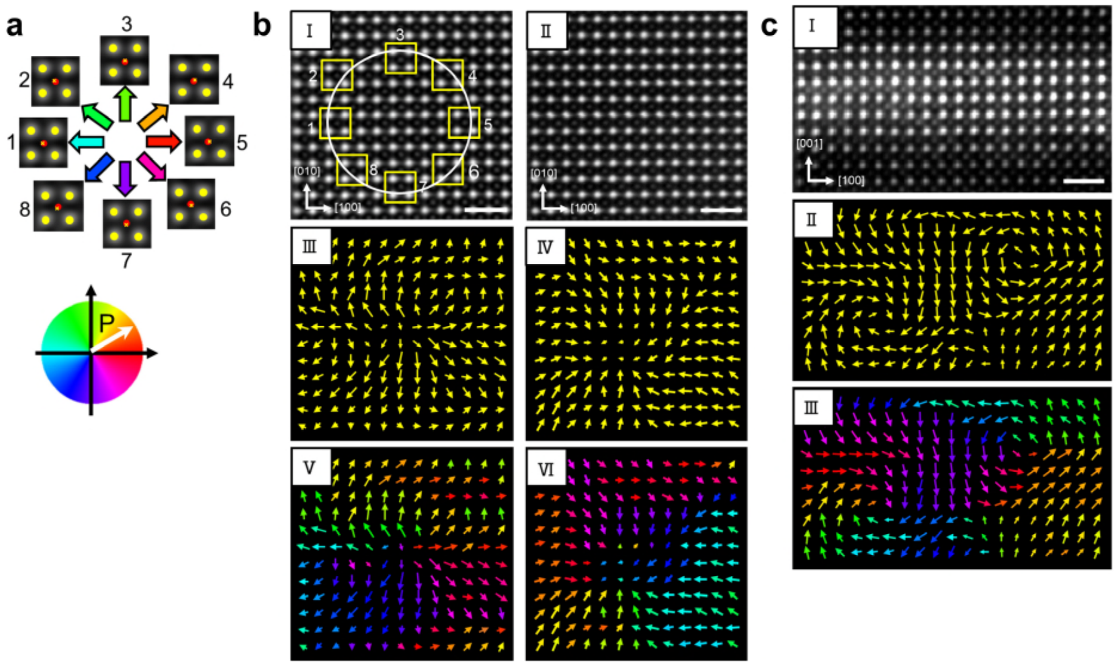

Fig. S6 | Method of extraction of polar displacement vectors from the HAADF-STEM image and characterization of solitons in a ($BFO_7/STO_4)_{10}$ superlattice. (**a**) Polar vector determination was performed on the Cs-corrected HAADF-STEM images using local A- and B-site sublattice offset measurements. The polar displacement vectors (denoted by arrows) were taken as the distance between each Fe atom (marked by filled red circle) and the mean position (marked by small yellow “×”) of the Fe’s four nearest Bi atoms (marked by filled yellow circles). Displacement vectors corresponding to local offsets between the A- and B-site sublattices were calculated by determining atomic positions by fitting each atom site by a spherical Gaussian using an algorithm in MATLAB. (**b**) Planar view HAADF-STEM images (I, II) and corresponding polar maps (III-VI). The arrow color in III denotes the angles between the direction of polarization and the horizontal, as shown in (**a**). Scale bar: 1 nm. (**c**) Cross-sectional HAADF-STEM image (I) and corresponding polar maps (II-III). Scale bar: 1 nm.

Next, we show evidence for the coexistence of multiple solitons in our superlattices. Based on in-plane and out-of-plane polar textures extracted from HAADF-STEM images in Fig. 2d-e of the main

manuscript, we can distinguish three characteristic topological structures, namely skyrmions, merons, and bimerons. The three-dimensional structure models of these solitons are shown in Fig. S7-I.

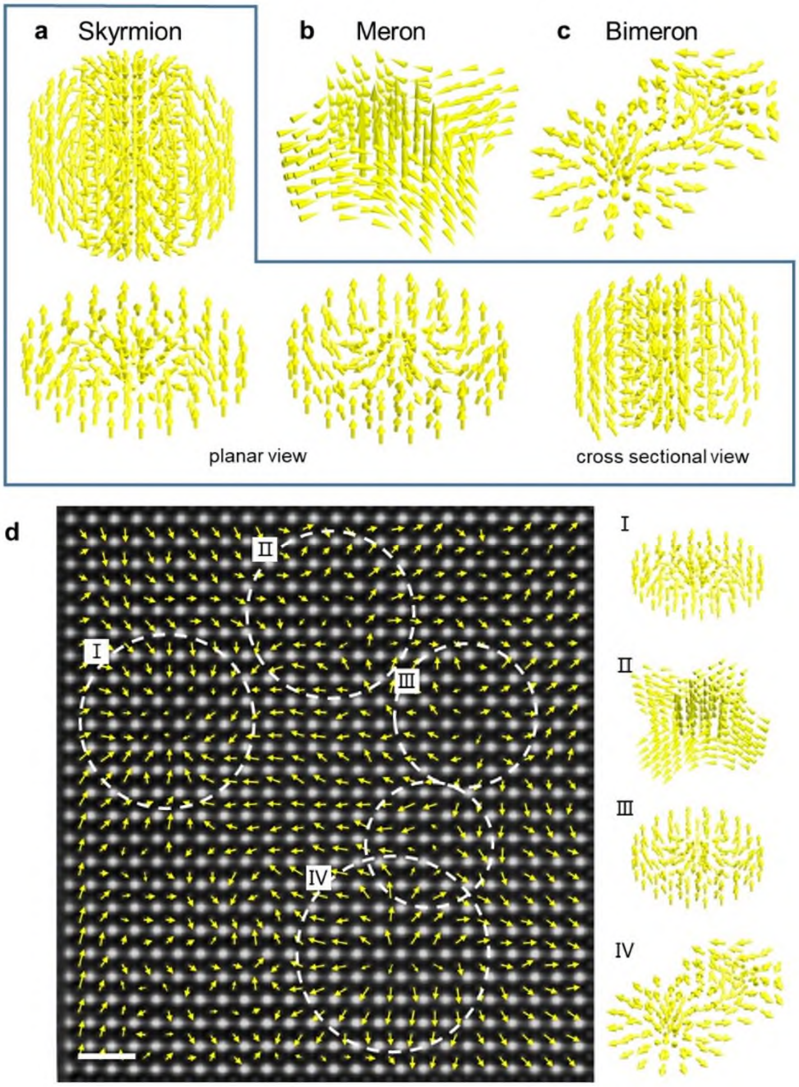

Fig. S7 | Coexistence of multiple soliton configurations. (**a-c**) 3D polar models of (**a**) skyrmion, (**b**) meron, and (**c**) bimeron. (**a**) shows the top plane (centre-convergent polar), bottom plane (centre-divergent polar) and cross sectional view of skyrmion. (**d**) In-plane polar map containing centre-convergent polar (I), anti-vortex (II), centre-divergent polar (III), and bimeron (IV) textures. Scale bar, 1 nm.

We now present evidence that the soliton structures are not simply localized to one region of the film. Fig. S7-II a shows a low-magnification planar view HAADF-STEM image of the superlattice. In three separate regions, the ring-like soliton structures are observed, as shown in Fig. S7-II b. Finally, the polar displacement mapping (Fig. S7-II c) of one such zoomed in region taken from III reveals polar soliton structures. Note that the soliton regions shown in Fig. S7-II are in a completely different region of the sample than the images presented in the main manuscript.

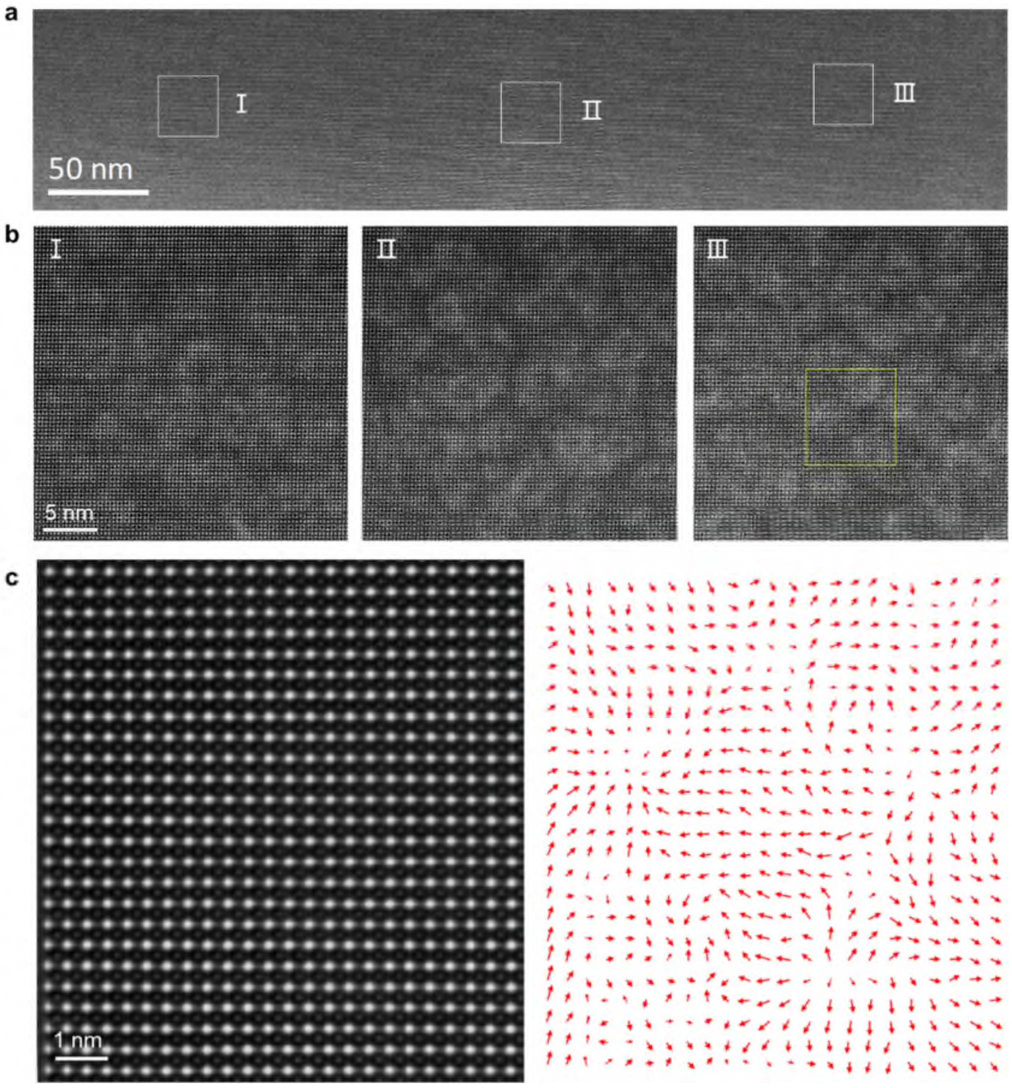

Fig. S7-II | (a), Low-magnification planar-view HAADF-STEM image of the BFO/STO superlattice. (**b**), Magnifications of areas I, II and III labelled in **a**, showing that the solitons formed at all locations. (**c**), Magnifications of area (marked by a yellow box) in **b** and corresponding polar map, showing the different solitons.

We also consider the distribution of the solitons in the cross-sectional STEM images. Figure S7-IIIa presents the cross sections HAADF-STEM images of the superlattice. Regions I, II and III are taken at different depths of the superlattice (notably, not at just the topmost surface BFO layer). In all regions, polar mapping (Fig. S7-III b) reveals that solitons are found.

Therefore, the results of Figs. S7 , I, II and II show that our $(BFO_7/STO_4)_{10}$ superlattices possess soliton polar structures throughout the film volume.

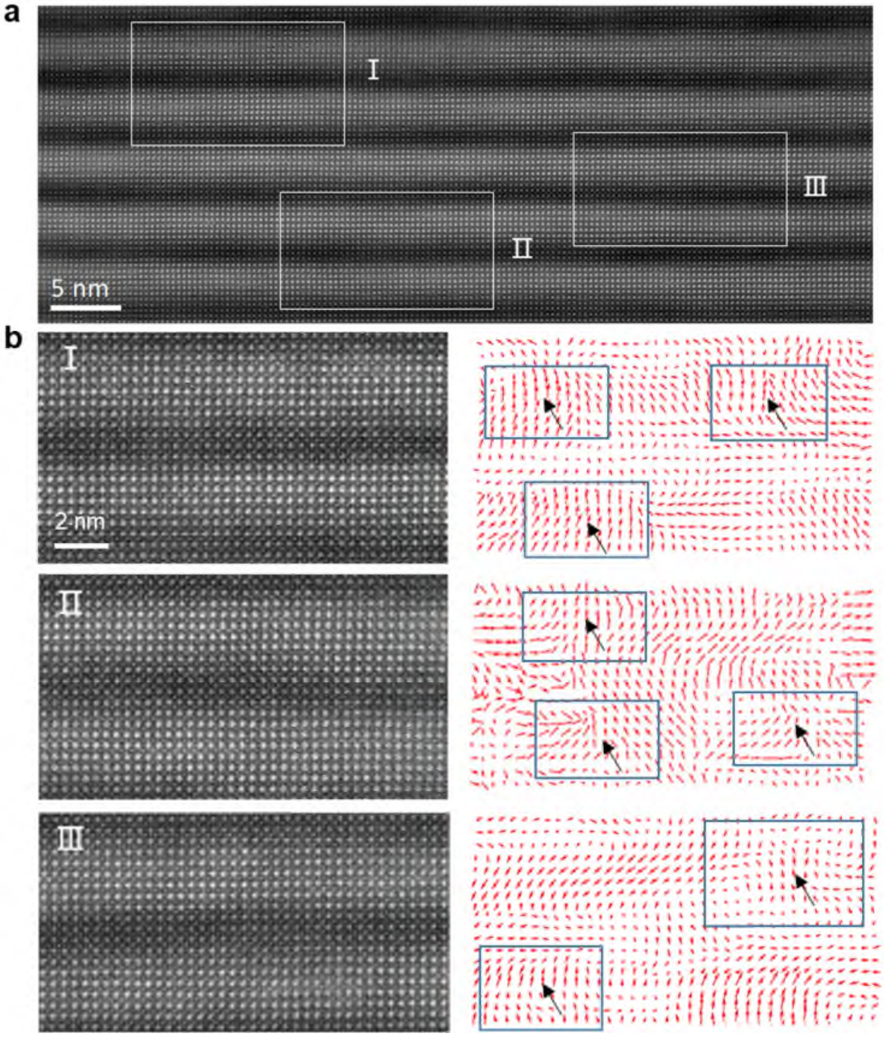

Fig S7-III (**a**), Low-magnification cross-section view of HAADF-STEM image of the BFO/STO superlattice. (**b**), Magnifications of areas I, II and III labelled in **a** and corresponding polar maps, showing the different solitons (marked by black arrows).

Further polarization mapping of BFO-STO layers

We have performed polarization mapping at larger scale. We find that although polarization also partially exists in the STO layer, the polarization in the BFO layer is still significantly greater than in the STO layer (Fig. S8c,f). The polarization map of Fig. 2e I (with a larger scale) is shown in Fig. S8d,e.

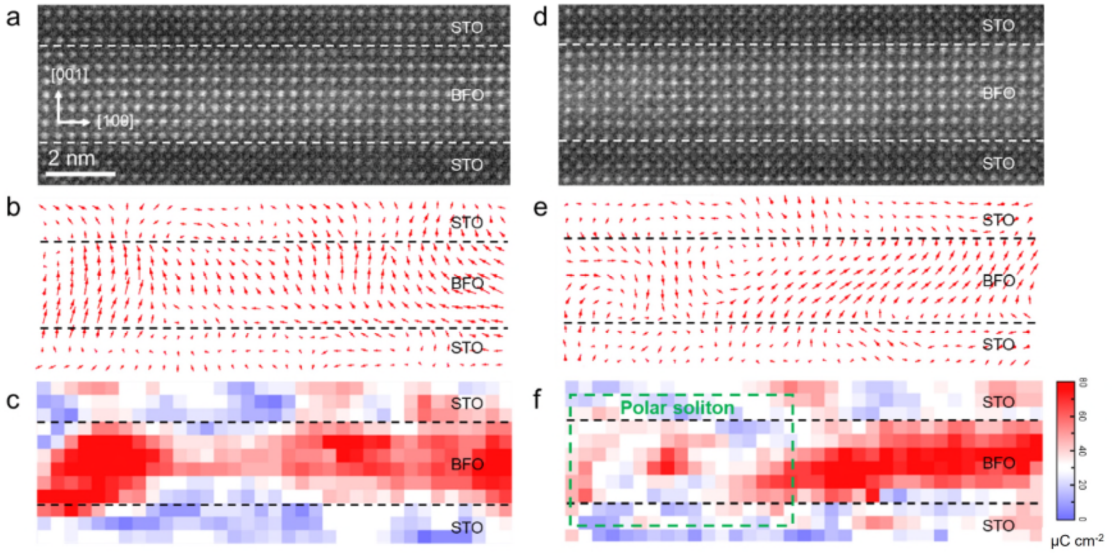

Fig. S8 | Polarization in BFO and STO layers. (**a,d**) HAADF-STEM images of BFO/STO layers. (**b,e**) Polar maps of HAADF-STEM images. (**c,f**) Polarization of BFO and STO layers. The colour maps represent the magnitude of polarization. Scale bar, 2 nm.

Supplementary Note 7. Geometric phase analysis (GPA) of strain states

To understand the stress state of our films at a more local scale, we have performed detailed GPA. Throughout the film, as a general rule, the BFO layers are under out-of-plane tensile strain, consistent with the in-plane compressive stress imposed by the LAO substrate. As shown in HAADF-STEM image and GPA analysis in Fig. S9, although the atomic interface between BFO and STO is clean (Fig. S9a), the tensile strain also partially exists in the STO layer, leading to induced polar order in the STO. The in-plane strain appears to be uniform in BFO and STO; the value of which depends on the lattice mismatch between the superlattice and the substrate.

Moreover, we observe periodic dislocations at the interface between the bottom BFO and LSMO, where the positions can be identified through the large in-plane strain variations (see Fig. S9b). Figure S9b-d show the in-plane strain ($\mathcal{E}_{xx}$), out-of-plane strain ($\mathcal{E}_{yy}$) and shear strain ($\mathcal{E}_{xy}$) distribution of the film. However note that no dislocations are evident in the BFO/STO superlattice layers. The in-plane strain in the middle BFO/STO area increases monotonically with distance from the substrate. At the same time, the out-of-plane strain and shear strain do not show a correlation with this change. Figure S9e is the relationship between average in-plane, out-of-plane, and shear strain with the distance from the substrate. We can see the BFO/STO layers in a state of "strain glass" where there is significant local strain variations on the nanometre scale, but this does not travel beyond a few nm. In other words, the BFO/STO layers host multiple local strain states which translates into a multiphase co-existence.

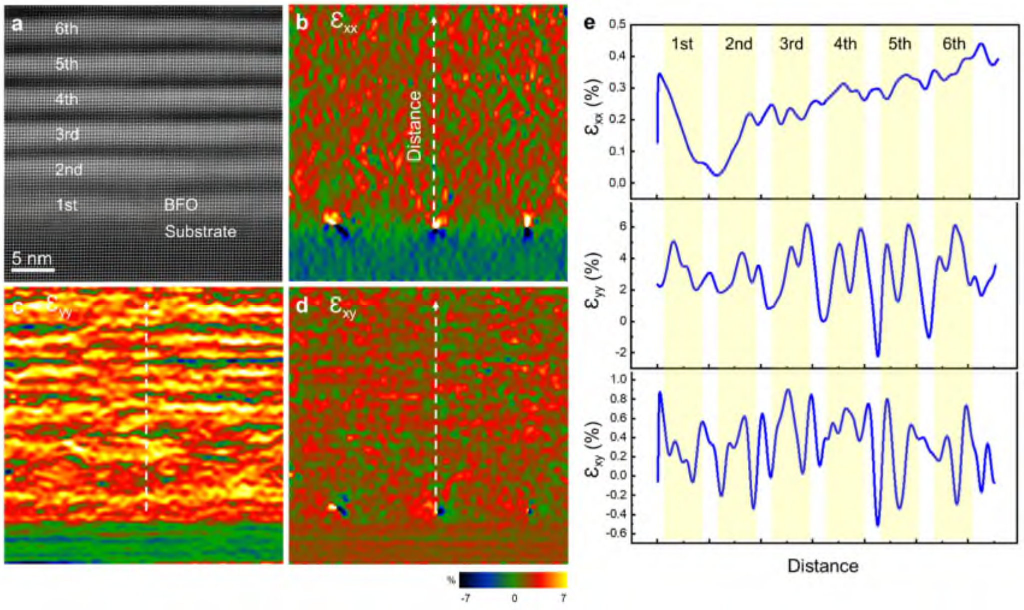

Fig. S9 | GPA of BFO/STO superlattice near the substrate-film interface. (**a**) HAADF-STEM image of BFO/STO film. (**b-d**) In-plane strain ($\mathcal{E}_{xx}$), out-of-plane strain ($\mathcal{E}_{yy}$) and shear strain ($\mathcal{E}_{xy}$) distributions of the film. (**e**) Average in-plane, out-of-plane, shear strain as a function of distance from substrate.

To make our argument further clear, we also show the strain and polarization map from a region taken in the middle of the BFO/STO superlattice (Fig. S10). There is a large range of strain variation, typically for such values we would expect the origins to be either dislocation formation or chemical disorder. We have already shown that there are no dislocations and the interfaces for the BFO/STO layers are sufficiently sharp to not allow such variations occurring over several nanometres. The only other possibility is that the local polarization pattern is changing dramatically, i.e., it is no longer constrained to the (001) (for T-like) or (111) (for R-like) BFO. It is assuming rotations that are not constricted to well-known crystallographic states (or strained phases) in order to form the solitons. As a result, we cannot use the traditional understanding of thin films applied to BFO that the film is in a single T or R phase. It houses a plethora of strain states due to the zoo of solitons formed.

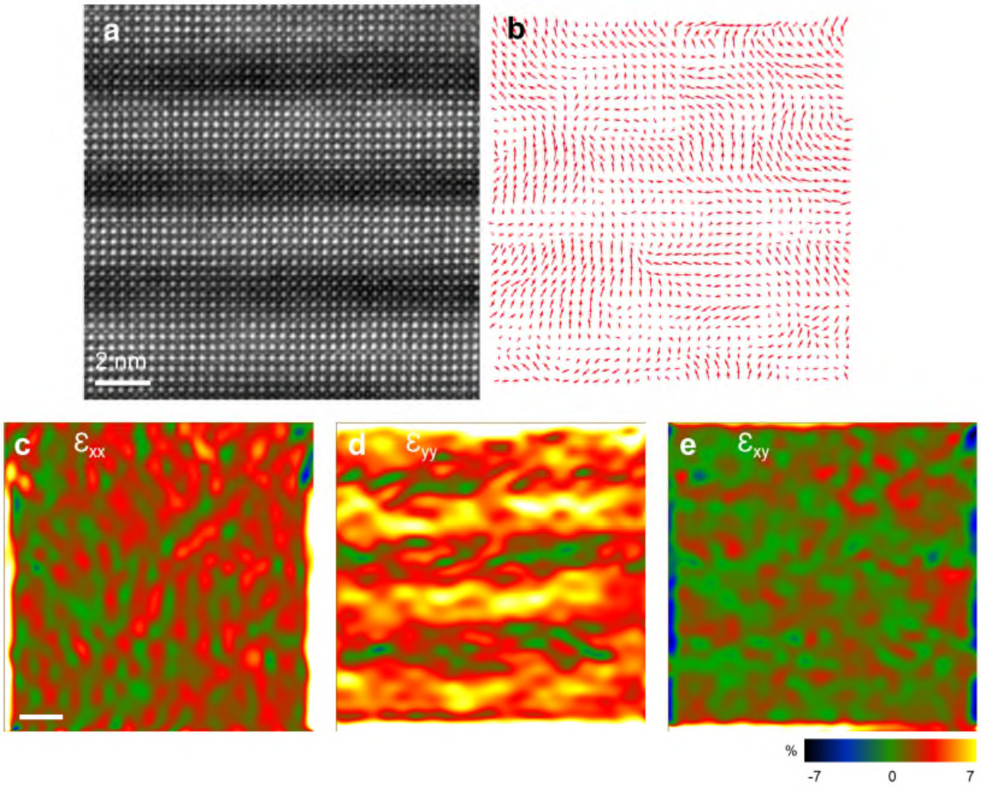

Fig. S10 | Strain and polarization analysis of BFO/STO film in the centre of the superlattice. (**a**) HAADF-STEM image in the middle layers of a $(7/4)_{10}$ BFO/STO superlattice. (**b**) Polar map of (**a**). (**c-e**) In-plane strain ($\mathcal{E}_{xx}$), out-of-plane strain ($\mathcal{E}_{yy}$) and shear strain ($\mathcal{E}_{xy}$) distributions of the superlattice. Scale bar, 2 nm.

Supplementary Note 8. Further phase-field simulation results

In this note, we present evidence from phase-field simulations of various other types of solitons in the BFO-STO superlattice system. Figure S11 shows the detailed polarization distribution on the top BFO layer in the BFO/STO superlattice. The magnitude of the in-plane polarizations is plotted, showing bubble-like patterns with high in-plane polarizations (Fig. S11a), like the soliton structures observed in the PTO/STO system. Meanwhile, the in-plane vector plot shows the co-existence of both center-convergent (indicated by a red circle) and center-divergent (indicated by a green circle) polarization patterns for the bubbles, consistent with the experimental observations by TEM (Fig. S7).

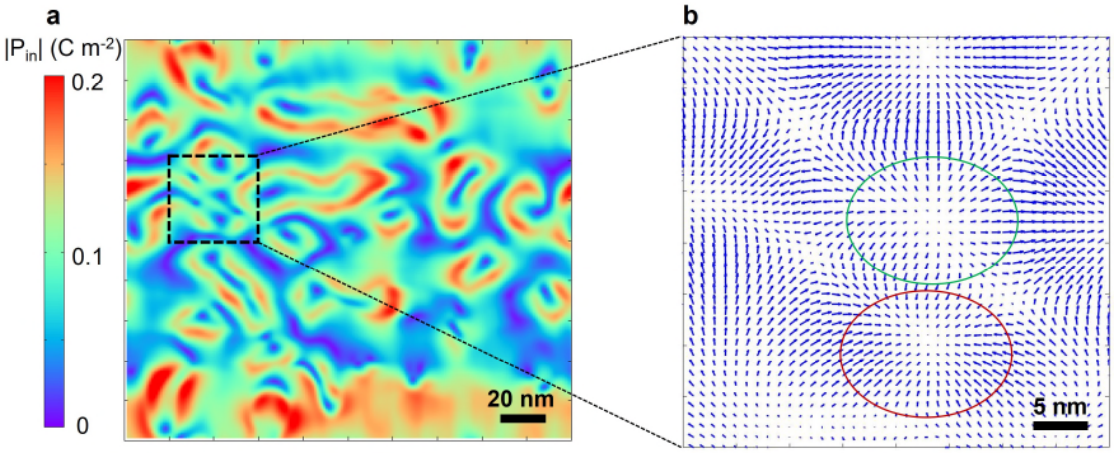

Fig. S11 | In-plane polarization distribution of the top BFO layer in the BFO/STO superlattice. (**a**) Planar view of polarization distribution, showing bubble-like regions with large values of in-plane polarization. (**b**) A region with both center-convergent (indicated by a red circle) and center-divergent (indicated by a green circle) polarization patterns from phase-field simulations.

We have also used phase-field simulations to show that applied bias to our superlattices can induce an increase in the soliton size. As shown in Fig. S12 under an applied electric field of -322 kV cm^{-1}, the size of the solitons for $(BFO_7/STO_4)_8$ superlattices can be increased from ~3 nm to ~10 nm for the small solitons, as marked by black arrow. It is also interesting to note that typically smaller solitons are more sensitive to external AC fields.

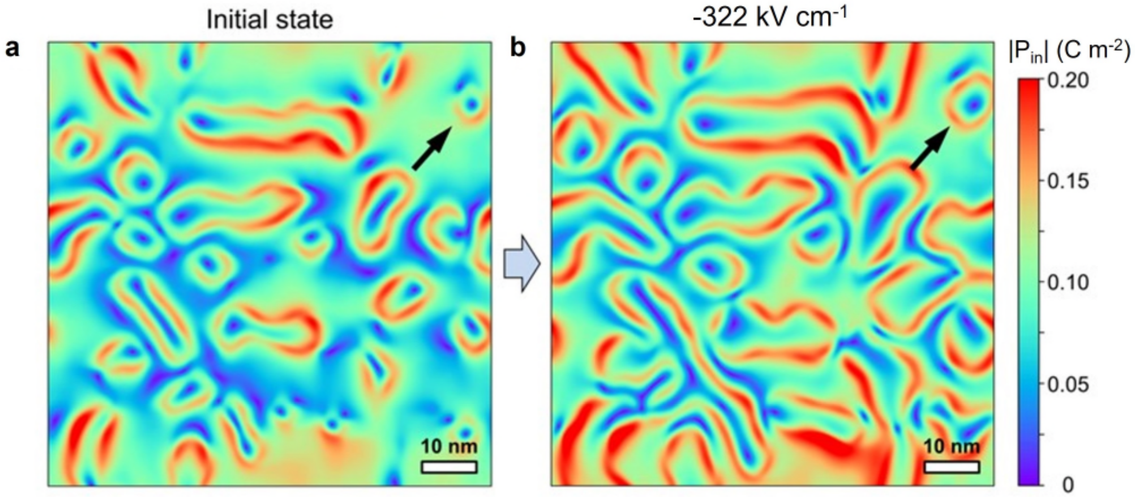

Fig. S12 | Planar view of the in-plane polarization for $(BFO_7/STO_4)_8$ superlattices calculated by phase-field simulations, showing the size-amplification effect under applied field. (**a**) Initial state without external field, (**b**) under an applied vertical electric field of -322 kV cm^{-1}. The size of the solitons can be increased with the applied field, for instance in the region denoted by the black arrow.

Finally, we use phase-field simulations to understand the thermodynamics of the phase stability in BFO/STO superlattices. The film thickness phase diagram of the BFO_n/STO_4 superlattices is first established (Fig. S13). When the BFO layer thickness is large ($n \geq 12$), 71° twin domains are formed, like the domain structure for BFO thin films on a STO substrate[3]. Upon decreasing the BFO layer thickness ($5 \leq n < 11$), the polar soliton state emerges, which is consistent with the experimental observations. When the BFO layer thickness is further reduced ($n \leq 4$), a monodomain state is the ground state, as has been shown in Fig. S2. To understand the energetics for the phase transitions, the individual energy densities vs. film thickness n was also plotted. The bulk energy density of the BFO/STO superlattices increases with the decrease of film thickness, due to the large reduction of the spontaneous polarization with thinner film. The elastic energy density, however, reduces dramatically which could compensate for the increase of the bulk energy density. The gradient and electric energy densities only change slightly with varying thickness. It can be concluded that the main driving force of the topological phase transitions is the competition between bulk and elastic energy densities.

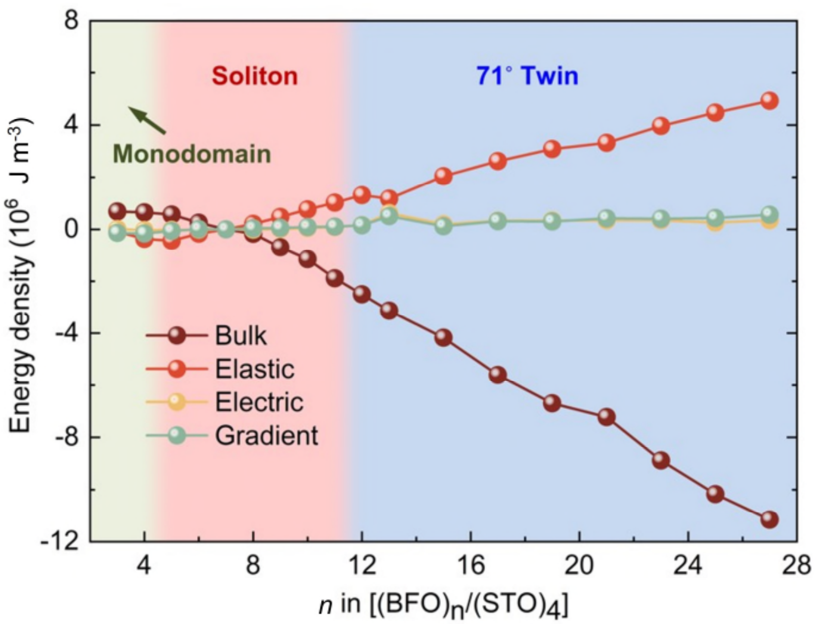

Fig. S13 | Film thickness phase diagram for BFO_n/STO_4 superlattices from phase-field simulations. The change of the individual energy densities with film thickness *n* (with respect to energy densities of BFO_7/STO_4 superlattice) is also shown.

Supplementary Note 9. Further effective Hamiltonian calculation results

In this note, we provide the result of effective Hamiltonian simulations for BFO film thicknesses similar to the experiment. The results (Fig. S.14) show similar vortex features at the same location as for higher thickness presented in the main manuscript, with similar Pontryagin's charge density.

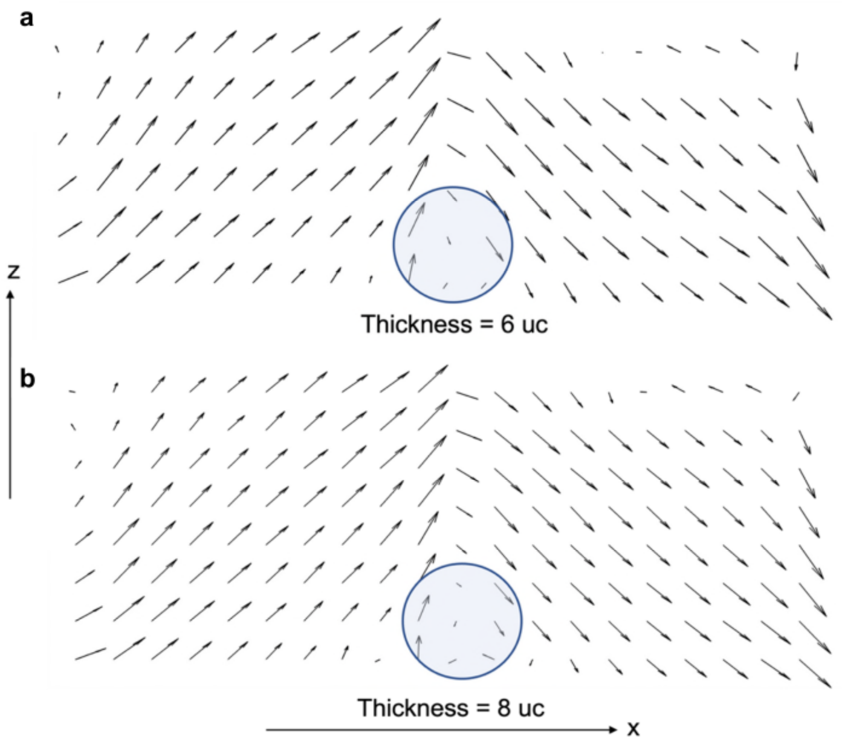

Fig. S14 | Polar mode in the x-z plane predicted by effective Hamiltonian calculation. Polar structure of BFO films with thickness of (**a**) 6 unit cells and (**b**) 8 unit cells. Vortex-like features are visible in both cases as highlighted by the circle.

References

1. Zhang, D. *et al.* Superior polarization retention through engineered domain wall pinning. *Nat. Commun.* **11**, 349 (2020).

2. Lichtensteiger, C. InteractiveXRDfit: A new tool to simulate and fit X-ray diffractograms of oxide thin films and heterostructures. *J. Appl. Crystallogr.* **51**, 1745–1751 (2018).

3. Nakashima, S. *et al.* Bulk photovoltaic effect in a $BiFeO_3$ thin film on a $SrTiO_3$ substrate. *Jpn. J. Appl. Phys.* **53**, 09PA16 (2014).
